# The synergistic effect of *Thymus vulgaris* essential oil and carvacrol with imipenem against carbapenem-resistant *Acinetobacter baumannii*: *in vitro*, molecular docking, and molecular dynamics studies

**DOI:** 10.3389/fphar.2025.1582102

**Published:** 2025-05-22

**Authors:** Saoussen Jilani, Mohamed Ferjeni, Kholoud Al-Shammery, Haya Rashid Mohammed AlTamimi, Malek Besbes, Salwa Ahmed Lotfi, Amr Farouk, Walid Ben Selma

**Affiliations:** ^1^ Department of Biology, College of Science, University of Ha’il, Ha’il, Saudi Arabia; ^2^ Laboratory of Analysis, Treatment and Valorization of Pollutants of the Environmental and Products, Faculty of Pharmacy, University of Monastir, Monastir, Tunisia; ^3^ Flavor and Aroma Chemistry Department, National Research Centre, Cairo, Egypt; ^4^ Higher Institute of Applied Sciences and Technology, University of Monastir, Mahdia, Tunisia

**Keywords:** *Thymus vulgaris*, carvacrol, GC/MS, antibiotic resistance, synergism, carbapenem resistant *Acinetobacter baumannii*, molecular docking, dynamic simulation

## Abstract

**Background:**

Carbapenem-resistant *Acinetobacter baumannii* (CRAB) is one of the most predominant causative agents of nosocomial infections, especially in the intensive care unit patients.

**Objective:**

The current study investigates the antibacterial activities of Tunisian *Thymus vulgaris* essential oil (Thyme-EO) alone and in combination with imipenem against CRAB.

**Methods:**

Thyme-EO antimicrobial activities were evaluated by disc diffusion and microdilution assays. Synergism between imipenem and Thyme-EO was determined by combined disc diffusion and checkerboard technique. The synergistic effect of the combined use of carvacrol and imipenem was evaluated by checkerboard assay. Interaction between the major compound identified by Gas Chromatography-Mass Spectrometry (GC/MS) of Thyme-EO and eight bacterial vital enzymes was analyzed by molecular docking and checked by molecular simulation for their stability.

**Results:**

According to GC/MS analysis, carvacrol (78.83%) was the major component. The inhibition zones’ diameter by Thyme-EO varied from 18 to 36 mm. Importantly, the values of minimum inhibitory concentration (MIC) and minimal bactericidal concentration (MBC) were of low level and ranged between 0.312 and 1.25 mg/mL. Interestingly, the MBC/MIC was equal to 1 for most tested bacterial strains, confirming a bactericidal effect of Thyme-EO. Combining imipenem and Thyme-EO diminished importantly the MIC of imipenem by 8- to 16-fold in the CRAB [fractional inhibitory concentration indexes (FICI) ˂ 0.5, synergy)]. Carvacrol showed antibacterial activities at low MIC levels of 64 and 128 μg/mL and advanced bactericidal effect justified by the MBC/MIC ratio, which was equal to 1 for most tested CRAB. Moreover, carvacrol interacts synergistically with imipenem against all bacterial isolates (FICI ˂ 0.5). The docking study demonstrated that carvacrol seemed to have high binding free energies (−8.1 kcal/mol) against D-alanine: D-alanine ligase (2ZDQ), which is implicated in the pathway of peptidoglycan’ biosynthesis. A 100-ns dynamic simulation investigation confirmed binding interactions and stability between carvacrol and the active residues of 2ZDQ.

**Conclusion:**

The current results demonstrated that carvacrol alone or combined with imipenem may constitute a promising opportunity as a novel strategy to treat infections caused by CRAB.

## 1 Introduction

The World Health Organization (WHO) has reported that most infectious agents are spreading highly and recommended the recognition and production of new bioactive molecules (WHO, 2024). The *Acinetobacter baumannii* strains, characterized as carbapenem-resistant, are considered critical to be fought due to their emergency. It was identified as a major source of hospital infections and classified as a critical priority pathogen due to its gene transfer abilities, severity of infections, and global impact, especially in low- and middle-income countries (WHO, 2024).

During the last 2 decades, because of widespread and various antibiotic therapeutics, Carbapenem-resistant *A. baumannii* (CRAB) strains have risen in hospital’s critical care units as a severe problem worldwide and have been extensively reported (WHO, 2024; Smith et al., 2007; Esterly et al., 2011). Accordingly, in the late 1970s, carbapenem antibiotics were the last therapeutic solution to combat these pathogens. Hence, *A. baumannii*, resistant to carbapenem, is included in the list of nosocomial pathogens cited by the WHO (WHO, 2024).

*Acinetobacter baumannii* strains have acquired several resistance mechanisms against carbapenem, for example, synthesis of enzymes inactivating antimicrobial agents, i.e., β-lactamase, vital decrease to attend bacterial targets (due to an advanced reduction of the outer membrane permeability triggered by low porin expression and elevated level of expression of multi-drug efflux pumps) (Manchanda et al., 2010; Santella et al., 2011), and different mutations affecting either targets or other cellular functions. Thus, for a single strain, these mechanisms of antimicrobial resistance may act alone or in combination (Rice, 2006).

Given that there is no identification and synthesis of new antibiotics against the carbapenem-resistant strains of *A. baumannii*, there is an urgent need to evaluate and validate novel approaches for treating infections caused by these microorganisms. One of these strategies, which appears of great importance, is investigating plant-derived substances for their antimicrobial activity used in traditional medicine (Rijo et al., 2024; Yilmaz et al., 2024; Ben Selma et al., 2023; Ben Selma et al., 2024a; Alibi et al., 2022; Ben Selma et al., 2024b). In this setting, the WHO prioritizes the research and development of potent therapeutic agents or adjuvants to antibiotics to fight resistant bacteria to antibiotics, mostly CRAB (WHO, 2024). Thus, a therapeutic strategy based on the simultaneous use of antibiotics and additive molecules could improve conventional antibiotics’ typical antibacterial activities, and the resistance phenotype of CRAB could change to a sensitive phenotype.

According to the literature, based on their multiple pharmaceutical bioactive components, medicinal plants have been extensively used as an essential source to synthesize diverse drugs for many decades. In this setting, our studies and others previously reported interesting effects of essential oils extracted from medicinal plants on a broad spectrum of pathogenic and multi-drug-resistant bacterial strains such as *Klebsiella pneumoniae*, *Salmonella enteritidis*, *Corynebacterium striatum,* and *Escherichia coli* (Ben Selma et al., 2023; Alibi et al., 2022; Alibi et al., 2020).

The genus *Thymus* is one of the most important parts of the Lamiaceae family, and it is represented by 214 species and 36 subspecies. Common thyme (*Thymus vulgaris* L., Lamiaceae) is one of these evergreen aromatic plants, which is grown in various parts of the world, mainly in the Mediterranean regions of North Africa (Stahl, and Saez, 2002), Europe, and Asia (Posgay et al., 2022). It has been an integral part of many cultures, satisfying various purposes in the culinary, aromatic, and medicinal domains (Santoro et al., 2007).

*Thymus vulgaris* is rich in essential oils, phenolic compounds, and flavonoids. These components give it powerful antioxidant, antimicrobial, and anti-inflammatory properties. Thymol and carvacrol, phenolic compounds the main constituents of *T. vulgaris* essential oil, are particularly effective in neutralizing reactive oxygen species and alleviating oxidative stress. These bioactive compounds have been shown to enhance immune function, reduce inflammation and exhibit antimicrobial activity against a broad spectrum of pathogens (Farhadi et al., 2024; El-Sayed and El-Sayed, 2020; Gavaric et al., 2015).

*Thymus vulgaris*, known for its medicinal properties, is used to treat wounds and respiratory ailments like bronchitis and laryngitis and addresses gastritis, cystitis, fluid retention, high blood pressure, heart issues, rheumatism, and arthritis (Kuete, 2017; Stahl and Saez, 2002; Basch et al., 2004; Li et al., 2019; Patil et al., 2021).

Carvacrol is a phenolic monoterpene, which is the major phytochemical compound found in the essential oils of *Thymus capitatus* (Ben Selma et al., 2023) and *Thymus algeriensis* (Ben Selma et al., 2024a) as we have previously reported. *Thymus capitatus* and *T. algeriensis* essential oils possess *in vitro* advanced bactericidal and synergistic activities with cefotaxime against Extended-Spectrum Beta-lactamases (ESBLs) producing *K. pneumoniae* hospital strains (Ben Selma et al., 2023), and synergism with colistin against multidrug-resistant Gram-negative bacterial isolates (Ben Selma et al., 2024a), respectively. These findings highlight the promising potential of carvacrol to be used in combination therapies and emphasize the importance of investigating synergistic interactions of this natural product with conventional antibiotics to effectively combat antimicrobial resistance.

The Federal Drug Administration recently approved carvacrol for human consumption (US Food and Drug Administration, 2024). The European Commission also permits certain phytochemicals in essential oils, including carvacrol, as food flavorings (Redondo-Blanco et al., 2020). Its hydroxyl group enhances its antibacterial potential, making carvacrol a potent bioactive compound (Ultee et al., 2002). Bioactive natural products show promising anti-Gram-negative bactericidal activities (Nazzaro et al., 2013). As antibiotic efficiency declines due to bacterial resistance, combining bioactive phytochemicals like carvacrol with antibiotics may enhance antimicrobial activity and reduce resistance.

Accordingly, because of the collective concern to generate new strategies to combat carbapenem-resistant bacteria, we focused in the current study to (1) determine the chemical composition of *T. vulgaris* essential oil by GC/MS; (2) evaluate the Tunisian *T. vulgaris* essential oil’ antimicrobial activities against CRAB strains; (3) determine whether combined adjuvant *T. vulgaris* essential oil/imipinem decreases the resistance of CRAB to imipenem; (4) assess antibacterial and synergistic effects of carvacrol with imipenem against CRAB strains; (5) identify by molecular docking for plausible interactions between major active substances of *T. vulgaris* essential oil and eight target comprising fundamental enzymes playing key roles in survival and biosynthesis of *A. baumannii*; (6) to check through a 100-ns dynamic simulation the stability and binding interactions between pharmacologically active substances of *T. vulgaris* essential oil and the active residues of targets.

## 2 Methods

### 2.1 Bacteriological analysis

#### 2.1.1 Isolates of bacteria

Twelve clinical strains of *A. baumannii* were obtained from the Laboratory of Microbiology of the University Hospital Taher Sfar, Mahdia, Tunisia. Bacterial isolates were identified using conventional methods, including Gram staining, colony morphology, and biochemical tests (Alibi et al., 2021; Tarchouna et al., 2013). *Escherichia coli* ATCC 25922 was used as an internal susceptible control strain.

#### 2.1.2 Antimicrobial susceptibility assay

Antimicrobial susceptibility testing was performed using a disk diffusion assay (Oxoid, USA), as previously recommended (EUCAST, 2024; CLSI, 2018). The results were interpreted according to the conventional guidelines (EUCAST, 2024). Bacterial strains were resistant to penicillins (ampicillin, piperacillin, ticarcillin), β-lactam (Ceftazidim, Cefoxitin, Cefixime, Cefepime, Aztreonam), cephems (cefotaxim, ceftazidime), β-lactam combination agent (Amoxicillin-clavulanic acid, Piperacillin-Tazobactam), carbapenems (ertapenem, imipenem, meropenem), aminoglycosides (gentamicin, tobramycin, amikacin), and quinolones and fluoroquinolones (nalidixic acid, ciprofloxacin).

#### 2.1.3 Determination of minimal inhibitory concentrations of imipenem

The MIC of imipenem (Sigma Aldrish, USA) against different selected bacterial strains was determined using the broth microdilution assay as a standard method (EUCAST, 2024; CLSI, 2018). Thus, sterilized Mueller Hinton II cation-adjusted broth in a sterile biological safety cabinet was distributed into 96-well round-bottom microplates (SARSTEDT AG and Co. KG, Numbrecht, Allemagne). Next, the imipenem was diluted by serial dilution at concentrations ranging from 2048 μg/mL to 0.012 μg/mL by a two-fold dilution method. These wells were inoculated with 100 µL 10^5^ CFU/mL of clinical bacterial isolates as well as *E. coli* ATCC 25922 as standard strain and incubated overnight at 37°C (Memmert, Germany). After incubation, the bacteria that showed visible growth at concentrations >4 μg/mL were considered resistant, as recommended by EUCAST (EUCAST, 2024). The assays were performed in triplicate to confirm the findings.

### 2.2 Essential oil of *Thymus vulgaris*

#### 2.2.1 Extraction of the *Thymus vulgaris* essential oil

The stems, leaves, and flowers of cultivated *T. vulgaris* were collected in a private farm in Chott Meriem, Sousse, a central region of Tunisia. The specific geographical coordinates are a latitude of 35° 56′ 17″ north and a longitude of 10° 33′ 18″ east. Plant samples were identified by Professor Fethia Harzallah-Skhiri, and voucher specimens were deposed in the herbarium of the High Institute of Biotechnology of Monastir, University of Monastir, Monastir, Tunisia, where they were assigned the code ‘Thvu.Lamiaceae63’. Then, the collected plant material was air-dried at ambient room temperature under shaded conditions.

The essential oil was extracted using the hydrodistillation method with the Clevenger apparatus. 100 g of sample and distilled water were boiled for 3 h. The oily phase was then condensed, and the essential oils were extracted, dried with anhydrous sodium sulfate, and stored in airtight glass vials sealed with aluminum foil at 4°C until analysis (Ben Selma et al., 2024b). The experiment was repeated three times.

#### 2.2.2 Phytochemical analysis of the *Thymus vulgaris* essential oil by gas chromatography-mass spectrometry

The analysis of the volatile compounds in *T. vulgaris* essential oil was carried out by using gas chromatography (Agilent 5890 II GC System, Folsom, CA, USA) combined with a mass spectrometer (Agilent 5977B GC/MSD) equipped with an HP-5MS fused silica capillary column (30 m, 0.25 mm i. d., 0.25 mm film thickness). As previously reported, the chromatographic separation was achieved (Ben Selma et al., 2023). Peaks were identified by comparing them to Wiley sixth. Edition mass spectral library, standards, and published data. Percentages of detected compounds were calculated using GC peak areas. Kovats index of each compound was determined using retention times of C6-C26 n-alkanes and compared to literature values (Adams, 2017).

#### 2.2.3 Evaluation of the antibacterial activities of *Thymus vulgaris* essential oil by disc diffusion assay

The antimicrobial activities of *T. vulgaris* essential oil were evaluated using a disc diffusion assay (Ben Selma et al., 2023). In a sterile biological safety cabinet, 5 µL of essential oil was added on sterile Whatman filter paper discs of a 6 mm diameter placed in the center of Petri plates previously spread with an overnight culture of each bacterial strain. Next, the Petri plates were incubated upside down at 37°C overnight. According to the diameter of the zone of inhibition around the disc, the *T. vulgaris* essential oil’ antibacterial activities were interpreted as previously reported: not sensitive if the diameter is ˂8 mm; sensitive if the diameter varies between 8 and 14 mm; very sensitive if the diameter ranging between 15 and 19 mm; extremely sensitive if the diameter ≥20 mm (Ben Selma et al., 2023).

#### 2.2.4 Determination of MICs and MBCs of *Thymus vulgaris* essential oil

*Thymus vulgaris* essential oil stock solutions were prepared in dimethyl sulfoxide (DMSO) (Sigma Aldrish, USA) at a final 100 mg/mL concentration. Then, these stock solutions were further diluted in the culture medium of Mueller Hinton II cation-adjusted broth to obtain working solutions with a final concentration of 40 mg/mL of *T. vulgaris* essential oil. It is important to note that the concentration of DMSO used in the *T. vulgaris* essential oil solutions experiments did not exceed 0.05%, which is non-toxic to bacteria (Cirino et al., 2023).

A microdilution susceptibility assay was used to determine the MICs of the selected *T. vulgaris* essential oil against all CRAB strains and *E. coli* ATCC 25922 used to control the quality of antibiotic susceptibility assays. The concentrations ranged from 40 to 0.078 mg/mL by two-fold serial dilutions. To determine the minimal bactericidal concentrations (MBC) from each well without growing bacteria, 5 µL were taken and spread on Mueller-Hinton agar (Biolife, Italiana, Italy) and incubated overnight to check if the bacterial growth inhibition was permanent or reversible (Ben Selma et al., 2023). The tests were achieved in triplicate.

#### 2.2.5 Combination *Thymus vulgaris* essential oil and imipenem

Simultaneous use of imipenem and *T. vulgaris* essential oil was evaluated using conventional assays, combined disk diffusion test, and checkerboard.

##### 2.2.5.1 Combined disk diffusion test

This test was applied as previously described (Ben Selma et al., 2023). For each CRAB strain, a bacterial suspension with a turbidity of 0.5 McFarland units was spread using a sterile swab over a Petri plate containing Mueller-Hinton agar (Biolife Italiana, Italy). Then, a commercial imipenem 10 µg (Oxoid, USA) disk was placed on the inoculated Mueller-Hinton agar plate and was impregnated with 5 μL of *T. vulgaris* essential oil. Finally, Petri plates were incubated overnight at 37°C.

A probable synergy between imipenem and the *T. vulgaris* essential oil was suggested if the difference between the two inhibition zones of essential oil in the presence or absence of imipenem is equal to or over 4 mm (Ben Selma et al., 2023).

##### 2.2.5.2 Checkerboard assay

Combined disk diffusion tests showing synergy in using imipenem and *T. vulgaris* essential oil were examined in checkerboard tests as previously described (Ben Selma et al., 2023). Briefly, in a safety cabinet, a microdilution assay of the imipenem and *T. vulgaris* EO was realized in Mueller Hinton II cation-adjusted broth (Sigma Aldrish, USA) and added to 96-well sterile plates according to conventional guidelines (EUCAST, 2024; CLSI, 2018). Then, the microplates were incubated under agitation at 37°C overnight. To confirm synergism between the two antimicrobial agents, the fractional inhibitory concentration indexes (FICI) were calculated as follows:

FICI = FIC essential oil + FIC imipenem; with

$$\text{FIC essential oil}=\frac{\text{MIC}\;\text{of}\;\text{essential}\;\text{oil}\;\text{in}\;\text{combination}}{\text{MIC}\;\text{of}\;\text{essential}\;\text{oil}\;\text{used}\;\text{alone}},$$

and

$$\text{FIC imipenem}=\frac{\text{MIC}\;\text{of}\;\text{imipenem}\;\text{in}\;\text{combination}}{\text{MIC}\;\text{of}\;\text{imipenem}\;\text{used}\;\text{alone}}$$

Based on FICI values, the interaction was considered as a synergism if FICI <0.5, as no interaction effect if 0.5 ≤ FICI ≤4, and as an antagonistic effect if FICI >4 (Ben Selma et al., 2023; Odds, 2003).

### 2.3 Antimicrobial activities of carvacrol

#### 2.3.1 Determination of MICs and MBCs of carvacrol

To evaluate the antimicrobial activities of the carvacrol, MICs were determined using the broth microdilution method in 96-well round-bottom microplates (SARSTEDT AG and Co. KG, Numbrecht, Germany) according to conventional guidelines (EUCAST, 2024; CLSI, 2018). The entire process was performed under sterile conditions in a sterile biological safety cabinet (BIOBASE, China). An antimicrobial stock solution of the natural product, carvacrol, was dissolved in 5% DMSO (Sigma Aldrich, USA). The concentrations of carvacrol were two-fold serial dilutions ranging from 1,000 to 1 μg/mL.

#### 2.3.2 Combination carvacrol and imipenem

The synergism between imipenem with carvacrol was evaluated using checkerboard assay (Ben Selma et al., 2023). Briefly, the antibiotic combination was cross-diluted in the wells of a 96-well microplate (SARSTEDT AG and Co. KG, Numbrecht, Germany) using Mueller Hinton II cation-adjusted broth (Sigma Aldrish, USA). The carvacrol was diluted vertically, with concentrations ranging from 250 to 1 μg/L. On the other hand, the imipenem was diluted horizontally, with concentrations ranging from 256 to 0.065 μg/L. The bacterial inoculum (10^5^ CFU/mL) was then added to the wells, after which the plates were incubated at 37°C for 24 h. The results were analyzed according to the FICI value, as stated below:

FICI = FIC imipenem + FIC carvacrol

$$\text{FIC imipenem}=\frac{\text{MIC}\;\text{of}\;\text{imipenem}\;\text{in}\;\text{combination}}{\text{MIC}\;\text{of}\;\text{imipenem}\;\text{used}\;\text{alone}},$$

and

$$\text{FIC carvacrol}=\frac{\text{MIC}\;\text{of}\;\text{carvacrol}\;\text{in}\;\text{combination}}{\text{MIC}\;\text{of}\;\text{carvacrol}\;\text{used}\;\text{alone}}$$

Results of calculated FICI are interpreted as follows: synergistic interaction (FICI ≤0.5), no interaction (0.5 < FICI ≤4), and antagonistic interaction (FICI >4) (Ben Selma et al., 2024a; Ben Selma et al., 2023; Odds, 2003).

### 2.4 Molecular docking

Ten bacterial enzymes were selected as binding targets based on their fundamental role in the vital functions of bacterial cells. These enzymes, including IV topoisomerase and DNA gyrase implicated in the replication of the DNA; D-alanine: D-alanine ligase, penicillin-binding protein 3, *A. baumannii* penicillin-binding protein 2, involved in the pathway of peptidoglycan synthesis; isoleucyl-tRNA synthetase, participated in protein synthesis; Sortase A, which anchors the majority of surface proteins; and Dihydrofolate reductase is an enzyme in the thymidine synthesis pathway.

The crystal structures of the following enzymes, isoleucyl-tRNA synthetase, DNA gyrase, dihydropteroate synthase, D-alanine: D-alanine ligase, IV topoisomerase, dihydrofolate reductase, penicillin-binding protein 3, Sortase A, *A. baumannii* penicillin-binding protein 2, and *A. baumannii* dihydroorotate dehydrogenase were obtained through the Protein DataBank (https://www.rcsb.org/, accessed on 17 August 2022, December 11–12, 2023, and 9 February 2024). These structures were used as receptors and identified with the following PDB IDs: 6LDK, 6FM4, 1AJ2, 2ZDQ, 7LHZ, 6XG5, 4KQR, 1T2P, 7ZG8, and 7UT5. The ligands used were downloaded from PubChem via http://pubchem.ncbi.nlm.nih.gov/on December 7 and 16, 2023. Pymol software ver. 2.5.1 was used to prepare the receptors by removing water, and co-crystallized ligands or ions may be found and then protonated. Meanwhile, the 3D structures of the ligands were optimized by Avogadro Software ver. 1.2.0. CB-DOCK2 was used for blind docking, accessed via http://clab.labshare.cn/cb-dock/php/on December 11–13 and 16, 2023, and February 11–12, 2024 as described previously (Liu et al., 2022). The top five cavities were submitted to AutoDock Vina for docking. Best-docked complexes were analyzed using Discovery Studio software, as described by Farouk and coworkers (Farouk et al., 2023).

### 2.5 Molecular dynamic simulation

The protein-ligand complexes were simulated using GROMACS 2021.1 with a forcefield of GROMOS96 54a7. Ligand topologies were generated with the PRODRG2 server. The MD simulation was performed at 300 K and 1 atm pressure for 100 ns with a salt concentration of 0.15 mol/L and a rectangular container using water molecules with a simple point charge (SPC). The stability of the complexes was evaluated by analyzing factors such as RMSD, RMSF, Rg, SASA, and hydrogen bonds. The results were plotted using the Xmgrace program (Farouk et al., 2023).

### 2.6 Statistical analysis

All experiments were replicated three times. The values were expressed as mean ± standard deviation (SD).

## 3 Results and discussion

### 3.1 Bacterial isolates and susceptibility testing

Twelve clinical strains of *A. baumannii*, isolated through conventional methods, were selected for this study based on their antibiotic resistance. Their antimicrobial susceptibility to standard antibiotics was assessed using a disc diffusion assay. These strains were classified as ultra-resistant *A. baumannii* isolates due to their resistance to the following antibiotic classes: aminoglycosides, carbapenems, cephalosporins, and fluoroquinolones. Consequently, they were identified as resistant to all antibiotics except colistin and tigecycline (Maragakis and Perl, 2008). The clinical isolates of CRAB were recovered from puncture samples (n = 5) and urine samples (n = 7).

### 3.2 Phytochemical composition of *Thymus vulgaris* essential oil

The chemical analyses of the *T. vulgaris* essential oil showed that carvacrol was the dominant constituent (78.83%). Other components were detected at very low levels, such as p-cymene (6.74%), γ-terpinene (3.08%), β-caryophyllene (1.78%), and linalool (1.58%) (Table 1). This characterizes our essential oil as a carvacrol chemotype. These results were not in the same linear with those previously published. Thus, a previous study indicated that p-cymene (29.52%) was the main constituent of *T. vulgaris* essential oil from Montenegro (Preljević et al., 2024). Moreover, two other studies reported that *T. vulgaris* essential oil from Italy was of thymol chemotype (68.02% and 44.4%, respectively) (Taglienti et al., 2022; Rinaldi et al., 2020). Another study showed that camphene (35.97%) was the major component of *T. vulgaris* essential oil from Egypt (Abd-Ellatif et al., 2022). The discrepancies in proportions of different components in different *T. vulgaris* essential oils samples of the current study and of these previous studies (Taglienti et al., 2022; Rinaldi et al., 2020; Abd-Ellatif et al., 2022) might be related to some parameters that took place at various stages, such as the climatic conditions, temperature, and humidity occurring during the growth of the plants (Lemos et al., 2017), the collecting period, phenological stages and different vegetation cycles (Moisa et al., 2019), the storage conditions of the samples, and the techniques of essential oil extraction (Llorens-Molina, and Vacas, 2017).

**TABLE 1:** Percentage of volatile components of *Thymus vulgaris* essential oil identified by GC/MS analysis.

Peak	RT	Area sum %	Compound
1	7,527	0,18	α-phellandrene
2	7,715	0,44	β-3-Carene
3	8,13	0,17	Camphene
4	9,002	0,13	1-Octen-3-ol
5	9,329	0,65	β-Myrcene
6	9,467	0,1	3-Octanol
7	9,721	0,11	α-Phellandrene
8	10,081	0,89	α−Terpeine
9	10,322	6,74	m-Cymene
10	10,445	0,24	Limonene
11	11,33	3,08	γ-Terpeine
12	12,207	0,13	α-Terpinolene
13	12,545	1,58	L-Linalool
14	14,516	0,82	1-Borneol
15	14,839	0,77	(−) -Terpinene-4-ol
16	15,233	0,12	α-Terpinyl acetate
17	15,41	0,08	(+)-Isodihydrocarvone
18	16,291	0,09	Nerol
19	17,149	0,07	Carvenone
20	17,463	0,07	*E*-Citral
21	17,902	0,79	*p-tert*-Butylpyridine
22	18,442	78,83	Carvacrol
23	20,185	0,98	Carvacryl acetate
24	21,474	1,78	*trans*-Caryophyllene
25	22,333	0,07	α-Humulene
26	23,351	0,06	Ledene
27	23,612	0,15	β-Bisabolene
28	24,417	0,12	*cis*-α-Bisabolene
29	25,491	0,67	Caryophyllene oxide

### 3.3 Antibacterial effects of *Thymus vulgaris* essential oil

For many decades, various plants have traditionally been a potential source for developing different drugs and therapeutic agents to treat various pathological disorders in humans (Cowan, 1999). Additionally, diverse plant compounds exerting antimicrobial properties, especially bactericidal effects against various microorganisms, were reported (Yilmaz et al., 2024; Ben Selma et al., 2023; Alibi et al., 2022; de Melo et al., 2023; Wei et al., 2023). Moreover, the antibacterial effects of different essential oils extracted from different medicinal plants on MDR *A. baumannii* strains *in vitro* were previously reported (Aleksic et al., 2014; Vázquez-Ucha et al., 2020). In this setting, the current study showed the highest values of the inhibition zone diameter (≥20 mm) with an average of 30.75 mm and bactericidal activity of *T. vulgaris* essential oil against all CRAB strains used (Table 2).

**TABLE 2:** Diameter of inhibition zone, MIC, and MBC of *Thymus vulgaris* essential oil. Synergistic effect of imipenem and *Thymus vulgaris* essential oil by disc diffusion against different *Acinetobacter baumannii* strains.

Strains	MIC (mg/mL)	MBC (mg/mL)	MBC/MIC	Diameter of inhibition zone by Thyme-EO (mm)	Diameter of inhibition zone by imipenem (mm)	Diameter of inhibition zone by the combined disc of imipenem and Thyme-EO (mm)
*A.baumannii*-100	0.625	1.25	2	20 ± 0.22	9 ± 0.2	30 ± 0.1
*A.baumannii*-101	0.312	0.312	1	29 ± 0.66	6 ± 0.1	40 ± 0.21
*A.baumannii*-102	0.625	0.625	1	30 ± 0.58	7 ± 0.13	34 ± 0.42
*A.baumannii*-103	1.25	1.25	1	34 ± 0.15	6 ± 0.14	47 ± 0.33
*A.baumannii*-104	1.25	1.25	1	33 ± 0.24	8 ± 0.32	36 ± 0.2
*A.baumannii*-105	0.625	0.625	1	28 ± 0.88	6 ± 0.12	34 ± 0.56
*A.baumannii*-106	0.625	0.625	1	28 ± 0.45	9 ± 0.21	32 ± 0.42
*A.baumannii*-107	0.625	0.625	1	29 ± 0.88	10 ± 0.13	40 ± 0.12
*A.baumannii*-108	1.25	2.5	2	20 ± 0.33	6 ± 0.22	30 ± 0.36
*A.baumannii*-109	0.625	0.625	1	34 ± 0.24	8 ± 0.31	40 ± 0.14
*A.baumannii*-110	0.625	0.625	1	33 ± 0.62	9 ± 0.18	36 ± 0.35
*A.baumannii-*111	1.25	1.25	1	31 ± 0.26	7 ± 0.15	36 ± 0.52
*E. coli* ATCC 25922	1.25	2.5	2	20 ± 0.34	24 ± 0.26	30 ± 0.32

*Thymus vulgaris* essential oil: Thyme-EO; minimal inhibitory concentration: MIC; minimal bactericidal concentration: MBC., number of replicates, n = 3.

The obtained results by the disc diffusion method were confirmed by those obtained by the MIC method (Table 2). Thus, the MIC values ranged between 0.312 and 1.25 mg/mL, with a median concentration of 0.8 mg/mL. Importantly, we found that the ratio MBC/MIC value was equal to one for ten of the twelve tested CRAB bacterial strains. These results argue for the strong bactericidal activities of the *T. vulgaris* essential oil at low concentrations. Thus, this finding supports the important bactericidal activities of *T. vulgaris* essential oil against CRAB. To our knowledge, this study is the first to report and demonstrate the advanced bactericidal effects of *T. vulgaris* essential oil against CRAB. Moreover, these results argue that this essential oil is a promising antibacterial agent for developing pharmaceutical drugs to treat severe infections caused by CRAB.

### 3.4 Screening for the interaction activity between *Thymus vulgaris* essential oil and imipenem

Table 2 shows the results obtained using a standard imipenem disc and *T. vulgaris* essential oil combination against all CRAB strains. Interestingly, the inhibition zones obtained by the combination test were more important than those obtained by using the essential oil alone with all CRAB strains. These results argued for a synergistic activity between *T. vulgaris* essential oil and imipenem.

Application of checkerboard assays on twelve CRAB strains showed that adding *T. vulgaris* essential oil to imipenem was interestingly associated with an important reduction of the antibiotic’s MIC. Moreover, synergism between imipenem and essential oil was obtained for seven tested CRAB strains (FICI ˂ 0.5, Table 3). Interestingly, the *T. vulgaris* essential oil reduced the imipenem MIC by 4- to 16-fold in the CRAB strains. A decrease in the imipenem MIC was also obtained in the susceptible *E. coli* ATCC 25922 strain, and a synergistic interaction was detected with this strain. The results of MICs and checkerboard values are presented in Table 3. The current finding is of great interest in restoring and enhancing the activities of imipenem against CRAB. This antibiotic is frequently used to treat human infections associated with *A. baumannii*.

**TABLE 3:** MIC of imipenem and *Thymus vulgaris* essential oil used alone. MIC of imipenem/*Thymus vulgaris* essential oil combinations. Values of FICI for the combinations against different *Acinetobacter baumannii* strains.

Strains	MIC imipenem (µg/mL)	MIC Thyme-EO (µg/mL)	MIC imipenem_Thyme-EO_** (µg/mL)	MIC Thyme-EO_imipenem_# (µg/mL)	FICI
*A.baumannii*-100	128	625	16	78	0.25 (Synergy)
*A.baumannii*-101	16	312	2	39	0.25 (Synergy)
*A.baumannii*-102	16	625	1	2	0.09 (Synergy)
*A.baumannii*-103	16	125	1	39	0.09 (Synergy)
*A.baumannii*-104	256	125	16	39	0.09 (Synergy)
*A.baumannii*-105	2048	625	64	2	0.09 (Synergy)
*A.baumannii*-106	2048	625	128	39	0.12 (Synergy)
*A.baumannii*-107	8	625	0.5	2	0.09 (Synergy)
*A.baumannii*-108	4	125	1	2	0.26 (Synergy)
*A.baumannii*-109	1,024	625	32	39	0.09 (Synergy)
*A.baumannii*-110	8	625	2	2	0.28 (Synergy)
*A.baumannii-*111	4	125	1	39	0.28 (Synergy)
*E. coli* ATCC 25922	0.25	125	0.03	39	0.12 (Synergy)

Minimal inhibitory concentrations: MIC; Thymus vulgaris essential oil: Thyme-EO; MIC, of imipenem in the presence of Thymus vulgaris essential oil; MIC, imipenemThyme-EO**; MIC, of Thymus vulgaris essential oil in the presence of imipenem: Thyme-EOimipenem#; fractional inhibitory concentration index: FICI.

In the current study, the synergistic effects obtained between imipenem and *T. vulgaris* essential oil against CRAB strains were of great interest to increase the susceptibility of these strains to one of more prescribed carbapenem antibiotic to treat infections related to these bacteria. Our results seemed to be in the same linear as earlier reports, which indicated synergistic action of the simultaneous use of antibiotics and essential oils against multi-drug resistance bacteria. Thus, some studies reported synergism in the following combinations: *Protium heptaphyllum* essential oil with amikacin (de Melo et al., 2023); *Tea tree* essential oil with amoxicillin (Wei et., 2023); *Syzygium aromaticum* essential oil and *Thymus zygis* essential oil with colistin (Vázquez-Ucha et al., 2020), and *Coriandrum sativum* essential oil with ciprofloxacin (Duarte et al., 2012).

### 3.5 Antibacterial activities of carvacrol

Carvacrol displayed significant inhibitory effects against all tested CRAB isolates, and the susceptible *E. coli* ATCC 25922 strain, with MICs varied between 64 and 128 μg/mL (Table 4). Our results were approximately similar to those previously reported by Cirino and collaborators, whose reported MIC values of carvacrol varied between 32 and 128 μg/mL (Cirino et al., 2023). However, the MICs of the current study are higher than those stated in another study by Aleksic Sabo and colleagues, whose reported MIC rates ranged between 7 and 28 μg/mL (Aleksic Sabo et al., 2021). These discrepancies between the results of these studies could be related to the technical details of the analysis used.

**TABLE 4:** MIC of imipenem and carvacrol used alone. MIC of imipenem/carvacrol combinations. Values of FICI for the combinations against different *Acinetobacter baumannii* strains.

Strains	MIC imipenem (µg/mL)	MIC carvacrol (µg/mL)	MIC imipenem_carvacrol_** (µg/mL)	MIC carvacrol_imipenem_# (µg/mL)	FICI
*A.baumannii*-100	128	64	4	4	0.09 (Synergy)
*A.baumannii*-101	16	64	1	2	0.09 (Synergy)
*A.baumannii*-102	16	64	1	2	0.09 (Synergy)
*A.baumannii*-103	16	128	1	2	0.07 (Synergy)
*A.baumannii*-104	256	128	4	2	0.03 (Synergy)
*A.baumannii*-105	2048	64	8	2	0.03 (Synergy)
*A.baumannii*-106	2048	64	4	2	0.03 (Synergy)
*A.baumannii*-107	8	64	1	2	0.15 (Synergy)
*A.baumannii*-108	4	128	1	2	0.26 (Synergy)
*A.baumannii*-109	1,024	128	4	4	0.03 (Synergy)
*A.baumannii*-110	8	64	1	2	0.15 (Synergy)
*A.baumannii-*111	4	128	0.5	4	0.15 (Synergy)
*E. coli* ATCC 25922	0.25	128	0.015	4	0.09 (Synergy)

Minimal inhibitory concentrations: MIC; MIC, of imipenem in the presence of carvacrol; MIC, imipenem_carvacrol_^**^; MIC, of carvacrol in the presence of imipenem: carvacrol_imipenem_#; fractional inhibitory concentration index: FICI.

Moreover, a notable observation in the current study is that there is no increased dosage requirement for MBCs compared to MICs, indicating no need for a higher concentration of carvacrol for bactericidal effects. Thus, the ratio MBC/MIC value was equal to one for all tested CRAB strains. This finding supports the advanced bactericidal activities of the natural product, carvacrol, at low concentrations. To the best of our knowledge, this study is the first to indicate the higher bactericidal effects of carvacrol against CRAB. Additionally, this outcome may represent an advantage to use carvacrol as a favorable antimicrobial molecule to treat severe infections associated with CRAB.

### 3.6 Synergism between carvacrol and imipenem

Based on the analysis of the checkerboard microdilution test results according to CLSI standard guidelines, the values obtained from which are listed in Table 2, FICI values in the combination of carvacrol plus imipenem in twelve isolates of CRAB and the susceptible *E. coli* ATCC 25922 strain were calculated in the range of 0.03–0.26. This finding argues for the synergistic interactions between carvacrol and imipenem against selected bacterial isolates. To our knowledge, our study is the first to report synergism between these two antimicrobial agents to combat CRAB.

It was established that natural products usually have weaker antibiotic activity than conventional antibiotics; consequently, it is difficult for them to effectively replace current antibiotics in clinical practice (Nazzaro et al., 2013). However, in the current study, the plant-derived antimicrobial compound carvacrol has synergistically enhanced the activity of imipenem against CRAB and susceptible *E. coli* ATCC 25922 strains. Thus, synergistic interaction between carvacrol and imipenem may allow for the combination to be as effective as the antibiotic alone, and while maintaining the use of commercial antibiotics, it lowers the MIC of both the antibiotic and the natural product. Moreover, the major finding of our study will offer the opportunity to develop a new innovative formula of therapeutic anti-infectious composed of a lower concentration of both agents which will give an important strategy as a new alternative to treat infectious diseases caused by ultra-resistant ESKAPE pathogens, as combinations with synergistic effects may reduce the probability of the emergence of bacterial resistance while having effective pharmacological results. Furthermore, it may involve a reduction in conventional antibiotic toxicity with fewer side effects compared to those derived from high doses of synthetic drugs. Additionally, this strategy will change the phenotype of multi-drug-resistant bacteria to sensitive bacteria of the selected conventional and frequently used antibiotics to treat severe infections associated with these pathogens. These findings reinforce this phytochemical compound’s potential as an adjunct therapy in treating infections caused by CRAB.

According to previous studies, the mechanism of action of carvacrol on bacterial strains is well-documented (Kachur, and Suntres, 2020; Nazzaro et al., 2013; Gill, and Holley, 2006). Due to its hydrophobic characteristics, carvacrol can insert itself into the fatty acid chains of cell membranes, disrupting membrane integrity and affecting the structure of porins and efflux pumps implicated in antibiotic resistance of bacteria (Zhang et al., 2018; Xu et al., 2008). This leads to an increase in membrane permeability, allowing the penetration of molecules such as imipenem at high levels to the cytoplasmic area, and this could justify the synergistic interaction between carvacrol and imipenem obtained in this study.

### 3.7 Molecular docking analysis

**FIGURE 1:**
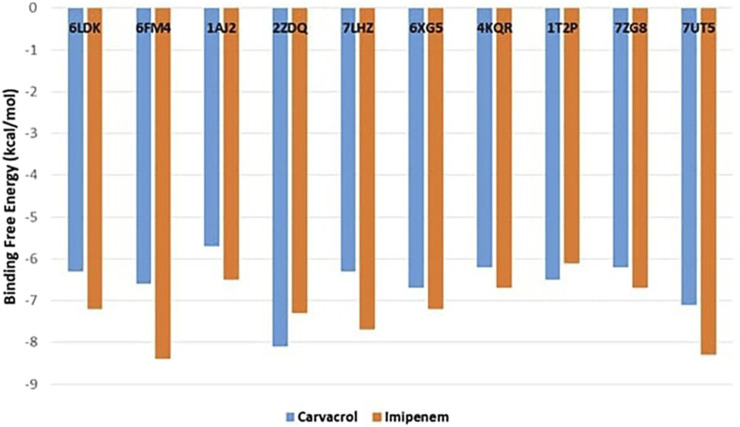
Binding free-energy values calculated through the molecular docking of the major Thymus vulgaris carvacrol volatile component and the bacterial key metabolic enzymes as receptors.

The current study applied the molecular docking approach to better understand how the major *T. vulgaris* volatile under investigation provides antibacterial activities. This carvacrol volatile component as a ligand was docked into vital enzymes involved in the biosynthesis and repair of cell walls, proteins, and nucleic acids (PDB IDs: 6LDK, 6FM4, 1AJ2, 2ZDQ, 7LHZ, 6XG5, 4KQR, 1T2P, 7ZG8, and 7UT5). Figure 1 displays the best poses obtained from the molecular docking analyses, revealing the binding affinities of the carvacrol ligand with the different bacterial enzymes. A lower ∆G indicates a more vital interaction between the receptor and the ligands. The carvacrol displayed higher binding affinities with high docking scores, ranging from −8.1 to −6.7 kcal/mol, compared to control (imipenem) towards the bacterial enzymes 2ZDQ, 4KQR, and 1T2P as shown in Figure 1, following by 6FM4 and 1T2P with −6.6 and −6.5 kcal/mol. Pencillin-binding proteins 3 of *Pseudomonas aeruginosa* (4KQR) and *A. baumannii penicillin-binding protein 2* (7ZG8) showed comparable docking results against the major volatile component carvacrol of *T. vulgaris* with −6.2 kcal/mol (Figure 1). Pencillin-binding proteins of *P. aeruginosa* (4KQR) and *A. baumannii* (7UT5) showed comparable docking results against the major volatile component carvacrol of *T. vulgaris* (Figure 1). For example, the same binding free energy was recorded for both proteins against carvacrol (−6.2 kcal/mol). Meanwhile, carvacrol showed the highest affinity against *A. baumannii* dihydroorotate dehydrogenase (7UT5). In conclusion, carvacrol showed potential docking scores, especially against 2ZDQ and 7UT5.

The previous findings proved a higher activity for the investigated oil compared to the volatiles of *Origanum compactum*, *Salvia officinalis*, and *S. aromaticum* extracted from east of Morocco and examined against DNA gyrase topoisomerase II and enoyl-acyl carrier protein reductase with a binding affinity ranging from −4.1 to −6.7 kcal/mol (Loukili et al., 2023). In the same line, both citronellal and terpinene-4-ol showed a lower binding energy toward DNA gyrase-B (PDB ID: 6F86) with −5.7 and −5 kcal/mol (Yuan and Hao, 2023). Also, the antibacterial activities of both *Origanum grossii* and *Thymus pallidus* essential oils were tested against topoisomerase enzyme (PDB ID: 1AJ6) with respect to their major components: carvacrol (−6.1 kcal/mol) and thymol (−5.7 kcal/mol), which are lower compared to the results of the present study (Zejli et al., 2023).

**FIGURE 2:**
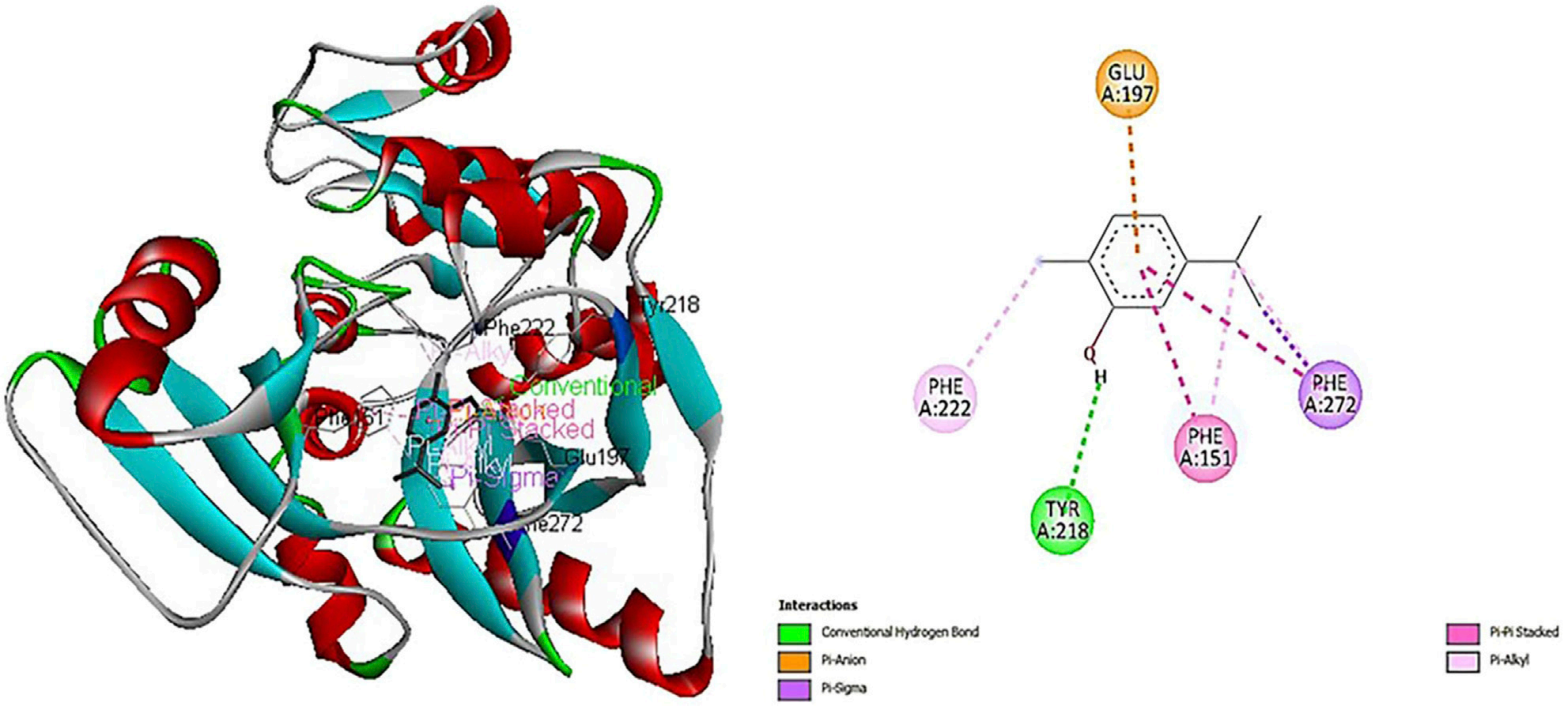
Interactions of carvacrol with D-alanine: D-alanine ligase (2ZDQ). Contact residues: LYS116 VAL 131 PHE151 LYS153 SER160 ILE163 ARG165 GLU189 LYS190 ALA191 LEU 192 VAL195 GLU197 TYR218 PRO221 PHE222 TYR223 LYS228 ASP270 PHE272 ASN281 GLU282. Center (X, Y, Z): 51, 16, 5, Docking size (X, Y, Z): 26, 17, 27.

**FIGURE 3:**
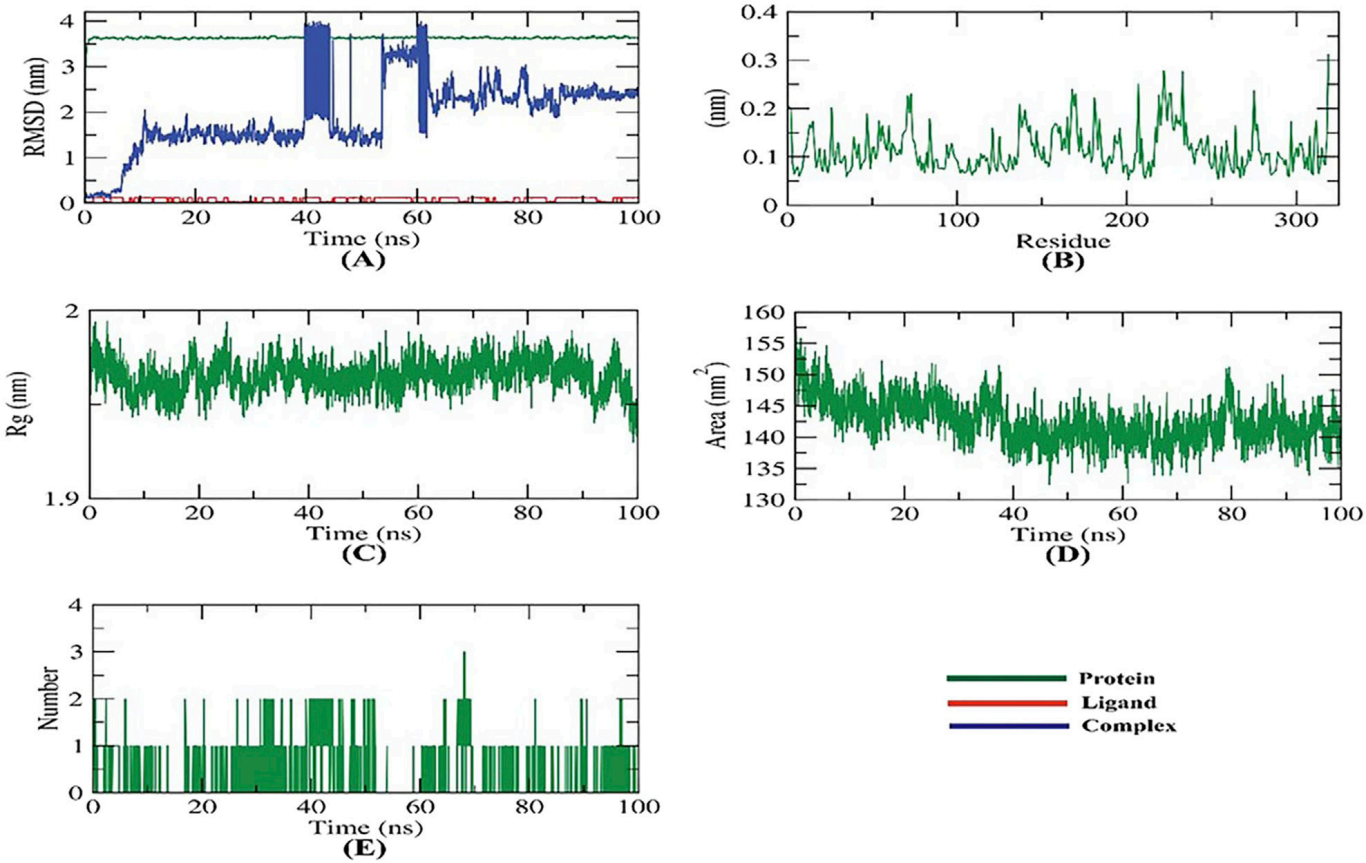
MD simulations of carvacrol-2ZDQ complex: **(A)** RMSD, **(B)** RMSF, **(C)** Rg, **(D)** SASA, and **(E)** H-bond analysis.

Figure 2 depicts how carvacrol interacts with the crystal structure of D-alanine: D-alanine ligase (PDB: 2ZDQ). This interaction exhibits the highest docking scores, as illustrated in Figure 2. The primary reason behind the higher binding affinity of carvacrol with 2ZDQ (−8.1 kcal/mol) is due to the conventional hydrogen bonding of the hydroxyl group of carvacrol (H-donor) to the OH-group of TYR A:218 (H-acceptor). Ligands behave more often as donors than acceptors among O–H⋯O interactions, which is consistent with the present findings. A distance of 2.5Å reveals a strong bond with free energy between −1.5 and −4.7 kcal/mol (Ferreira de Freitas and Schapira, 2017). Other interactions were also observed, including π-anion, π-σ, π-π stacked, and π-alkyl electrostatic and hydrophobic interactions (Figure 3). The only electrostatic bond of the π-anion type is formed between the negative carboxylic group of GLU A:197 and the π-orbitals of carvacrol. An anion-π interaction is established between an anion and an electro-deficient aromatic group that exhibits interesting synergic effects called cooperativity with the conventional hydrogen bonds, as Plais and collaborators reported (Plais et al., 2023).

The π- σ hydrophobic interaction from the C-H of carvacrol to π-orbitals of PHE A:272. Half of all Phe rings were involved as acceptors in most prominent CH…π-interactions between aliphatic C-H donors and aromatic π–acceptors (Brandl et al., 2001). The current study also revealed that carvacrol alkyl groups interact with aryl-containing amino acids like PHE A:151, 222, and 272 through their π-orbitals (Figure 2). Additionally, a π-π stacked interaction is the attractive force between aromatic rings due to the presence of π-electron clouds like the π-orbitals of PHE A:151 and 272 and carvacrol. π-π stacking is a noncovalent interaction between aromatic rings, important in biological recognition and biomolecular structure organization. It provides a binding energy of about 2–3 kcal/mol (Brylinski, 2018). Electron-deficient rings stack better than electronic ones. Aliphatic-aromatic and aromatic-aromatic edge-to-face contacts have similar stabilization levels (Ferreira de Freitas and Schapira, 2017).

The pathway of peptidoglycan biosynthesis inhibitors has historically been proven to be one of the most important targets for antibiotic development (Sarkar et al., 2017). This is due to the role of peptidoglycan in maintaining cell structure and rigidity, which allows the bacteria to survive in a hypotonic environment and protect against osmotic lysis. Thus, the inhibition of peptidoglycan assembly induces cell lysis and death of bacterial strains. In this setting, based on the higher binding affinity of carvacrol with D-alanine: D-alanine ligase (PDB: 2ZDQ) (−8.1 kcal/mol) the enzyme implicated in the earlier stage of biosynthesis of peptidoglycan demonstrated in the current study, we suggest that interaction of carvacrol with 2ZDQ could inhibit the pathway of peptidoglycan biosynthesis and lead to cell lysis and death of bacteria.

### 3.8 Molecular dynamic simulation

Molecular dynamics simulations were conducted for 100 ns to study the effect of carvacrol binding on D-alanine: D-alanine ligase (2ZDQ), a target protein. The simulations examined the modeled structures of the protein, and various computations were performed, including structural, dynamic, and thermodynamic analyses of the observed trajectory. Various studies, including backbone RMSD, RMSF, Rg, SASA, H-bond, and MM/PBSA, were conducted to assess the protein’s structural behavior in both bound and unbound states.

The protein-ligand complex’s stability in the presence of the receptor and ligand-bound state was determined using RMSD (root-mean-square deviation) to calculate the dynamic movements of atoms and conformational changes of backbone atoms. Carvacrol had very low RMSD compared to 2ZDQ, indicating higher stability with no significant variations in both. The RMSD of the complex fluctuated before displaying stability, being unstable for approximately 10 ns and from about 40 to 60 ns (Figure 3A). The more stable protein structures have lower RMSD values and *vice versa* (Farouk et al., 2023).

During a simulation, the residues’ RMSF (root mean square fluctuation) was observed to measure their fluctuation from their average position. The number of residues indicates the flexibility of the proteins. Figure 3B shows the fluctuation is neutral, meaning the ligand binding does not affect the residue positioning. The protein compactness, as measured by the radius of gyration (Rg), varies depending on ligand coupling. Lower fluctuation over time indicates a more stable and compact system (Iskineyeva et al., 2022). The Rg of the 2ZDQ-carvacrol complex was found to be slightly lower than in the initial period (Figure 3C).

The study analyzed how the 2ZDQ-carvacrol complex interacts with solvents in its surroundings. The solvent-accessible surface area (SASA) was used to predict any conformational changes that may occur after the binding of complex components. It is worth noting that the surface area of 2ZDQ decreased during the 100 ns simulation shown in Figure 3D, while the SASA value remained constant. The protein-ligand combination must create hydrogen bonds for the structure to remain stable. The research revealed the formation of one to two hydrogen bonds between the most significant protein conformations and the ligand, as illustrated in Figure 3E.

**FIGURE 4:**
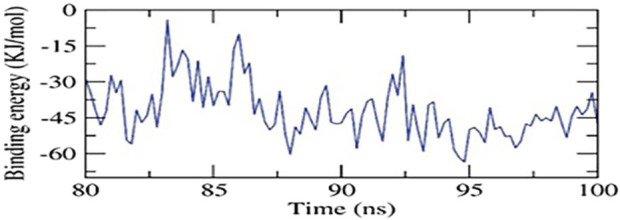
MM-PBSA study of the 2ZDQ-carvacrol complex.

The study predicted binding free energies for receptor-ligand complex systems using MD simulations with the MM/PBSA technique. It analyzed 2ZDQ-carvacrol’s binding affinity profiles to determine the optimal inhibitory action’s selectivity. The MM/PBSA method was used to calculate the binding free energy of the final 20 nanoseconds of the MD production run. The results showed that 2ZDQ protein had a binding free energy of −41 kJ/mol with carvacrol (Figure 4).

## 4 Conclusion

The current study is the first to evaluate the antibacterial activities of Tunisian *T. vulgaris* essential oil and its major compound carvacrol used alone and their use as an imipenem adjuvant; moreover, it demonstrated the synergistic activities of the combined use of these natural phytochemical antimicrobial agents against CRAB. The molecular docking showed the higher affinity of *T. vulgaris* essential oil major component’ carvacrol toward the vital enzyme D-alanine: D-alanine ligase implicated in the pathway of peptidoglycan’ biosynthesis of the CRAB. Furthermore, the 100ns dynamic simulation investigation confirmed the binding interactions and stability between carvacrol and the active residues of the vital enzyme D-alanine: D-alanine ligase. The current finding may be a promising therapeutic strategy as an alternative in developing a potential bactericidal treatment against CRAB. Further studies will be needed to evaluate the *in vivo* use to assess the safety and effectiveness of the combined consumption of carvacrol and imipenem to translate these findings into practical applications.

## Data Availability

The original contributions presented in the study are included in the article/Supplementary Material, further inquiries can be directed to the corresponding author.
