# Supplementary material for: The synergistic effect of Thymus vulgaris essential oil and carvacrol with imipenem against carbapenem-resistant Acinetobacter baumannii: in vitro, molecular docking, and molecular dynamics studies
Source: Front Pharmacol. 2025 May 22;16:1582102. doi: 10.3389/fphar.2025.1582102 (PMC12137262; doi:10.3389/fphar.2025.1582102)
Supplement: Supplementary file 1 [file Table1.doc]

Receptors/Proteins used in the current study were downloaded through [<https://www.rcsb.org/>], according to the following links:

6ldk: <https://www.rcsb.org/structure/6ldk>

6fm4: <https://www.rcsb.org/structure/6FM4>

1aj2: <https://www.rcsb.org/structure/1AJ2>

2zdq: <https://www.rcsb.org/structure/2ZDQ>

7lhz: <https://www.rcsb.org/structure/7LHZ>

6xg5: <https://www.rcsb.org/structure/6XG5>

4kqr: <https://www.rcsb.org/structure/4KQR>

1t2p: <https://www.rcsb.org/structure/1T2P>

7zg8: <https://www.rcsb.org/structure/7ZG8>

7ut5: <https://www.rcsb.org/structure/7UT5>

| **Code** | **Description** | **Organism** | **Expression System** |
| --- | --- | --- | --- |
| 6LDK | Isoleucyl-tRNA synthetase from *Candida albicans* | [*Candida albicans* SC5314](https://www.rcsb.org/search?q=rcsb_entity_source_organism.taxonomy_lineage.name:Candida albicans SC5314) | [*Escherichia coli*](https://www.rcsb.org/search?q=rcsb_entity_host_organism.ncbi_scientific_name:Escherichia coli) |
| 6FM4 | *S. aureus* Gyrase | [*Staphylococcus aureus* subsp. *aureus* N315](https://www.rcsb.org/search?q=rcsb_entity_source_organism.taxonomy_lineage.name:Staphylococcus aureus subsp. aureus N315) | [*Escherichia coli*](https://www.rcsb.org/search?q=rcsb_entity_host_organism.ncbi_scientific_name:Escherichia coli) |
| 1AJ2 | *E. coli* dihydropteroate synthase | [*Escherichia coli*](https://www.rcsb.org/search?q=rcsb_entity_source_organism.taxonomy_lineage.name:Escherichia coli) | - |
| 2ZDQ | D-Alanine:D-Alanine Ligase | [*Thermus thermophilus*](https://www.rcsb.org/search?q=rcsb_entity_source_organism.taxonomy_lineage.name:Thermus thermophilus) | [*Escherichia coli*](https://www.rcsb.org/search?q=rcsb_entity_host_organism.ncbi_scientific_name:Escherichia coli) |
| 7LHZ | *K. pneumoniae* Topoisomerase IV | [*Klebsiella pneumoniae* 342](https://www.rcsb.org/search?q=rcsb_entity_source_organism.taxonomy_lineage.name:Klebsiella pneumoniae 342) | [*Escherichia coli*](https://www.rcsb.org/search?q=rcsb_entity_host_organism.ncbi_scientific_name:Escherichia coli) |
| 6XG5 | *Escherichia coli* dihydrofolate reductase | [*Escherichia coli* K-12](https://www.rcsb.org/search?q=rcsb_entity_source_organism.taxonomy_lineage.name:Escherichia coli K-12) | [*Escherichia coli* BL21](https://www.rcsb.org/search?q=rcsb_entity_host_organism.ncbi_scientific_name:Escherichia coli BL21) |
| 4KQR | Penicillin-binding protein 3 | [*Pseudomonas aeruginosa* PAO1](https://www.rcsb.org/search?q=rcsb_entity_source_organism.taxonomy_lineage.name:Pseudomonas aeruginosa PAO1) | [*Escherichia coli* BL21(DE3)](https://www.rcsb.org/search?q=rcsb_entity_host_organism.ncbi_scientific_name:Escherichia coli BL21(DE3)) |
| 1T2P | Sortase A | [*Staphylococcus aureus*](https://www.rcsb.org/search?q=rcsb_entity_source_organism.taxonomy_lineage.name:Staphylococcus aureus) | [*Escherichia coli*](https://www.rcsb.org/search?q=rcsb_entity_host_organism.ncbi_scientific_name:Escherichia coli) |
| 7ZG8 | Penicillin-binding protein 2 | [*Acinetobacter baumannii*](https://www.rcsb.org/search?q=rcsb_entity_source_organism.taxonomy_lineage.name:Acinetobacter baumannii) | [*Escherichia coli*](https://www.rcsb.org/search?q=rcsb_entity_host_organism.ncbi_scientific_name:Escherichia coli) |
| 7UT5 | Dihydroorotate dehydrogenase | [*Acinetobacter baumannii*](https://www.rcsb.org/search?q=rcsb_entity_source_organism.taxonomy_lineage.name:Acinetobacter baumannii) | [*Escherichia coli* BL21](https://www.rcsb.org/search?q=rcsb_entity_host_organism.ncbi_scientific_name:Escherichia coli BL21) |

Ligands used in the current study were downloaded from the following address <https://pubchem.ncbi.nlm.nih.gov/> :

Carvacrol: <https://pubchem.ncbi.nlm.nih.gov/compound/Carvacrol>

Imipenem: <https://pubchem.ncbi.nlm.nih.gov/compound/Imipenem>
